# The Effect of Tamsulosin, an Alpha-1 Receptor Antagonist as a Medical Expelling Agent in Success Rate of Ureteroscopic Lithotripsy

**DOI:** 10.5812/numonthly.12836

**Published:** 2013-12-12

**Authors:** Ali Asghar Ketabchi, Soha Mehrabi

**Affiliations:** 1 Department of Urology, Faculty of Medicine, Kerman University of Medical Sciences, Kerman, IR Iran

**Keywords:** Ureteroscopy, Lithotripsy, α-1A-specific Blocker, Tamsulosin, Ureteral Calculi

## Abstract

**Background:**

Tamsulosin is an α-1A-specific blocker which induces selective relaxation of ureteral smooth muscle with subsequent inhibition of ureteral spasms and dilatation of the ureteral lumen and facilitates stone expelling.

**Objectives:**

In this study we aimed to assess the efficacy of tamsulosin for improving the success rate of ureteroscopic lithotripsy (URS) for lower ureteral stones.

**Patients and Methods:**

In a prospective study by a randomized controlled clinical trial, which was performed from June 2008 to December 2010, we enrolled one hundred and forty-two subjects and eventually 102 patients completed the clinical trial. All the patients underwent ureteroscopic lithotripsy with the pneumatic wolf lithotripsy. The patients were randomly divided into 2 groups: the study group including 52 patients, received tamsulosin with our traditional treatment (hydration and analgesic when required), and the control group with 50 patients who received placebo with traditional treatment. The number of colic episodes, lower urinary tract symptoms, analgesic dosage, and days of spontaneous passage of the stones through the ureter were recorded in a diary after lithotripsy.

**Results:**

The results showed that tamsulosin treatment group had low expulsion time (P = 0.011), low urinary tract symptoms, least analgesic needs and low adverse effects, all with statistically significant differences comparable with the control group (P < 0.05).

**Conclusions:**

Administration of α-1A-specific blocker reduced analgesic dosage and colic episodes and rate of adverse effects after ureteroscopic lithotripsy of lower ureteral stones and decreased gravel expulsion time after URSL.

## 1. Background

The urolithiasis is a common and increasing condition. The global mankind prevalence of urinary tract stones has been estimated to be between 2% to 20% and afflicting 13% of men and 7% of women.

The life time recurrence rate is approximately 50%, and 20% of whole urinary stones are ureteral stones, where 70% of these ureteral stones are located in the distal portion of the ureters and most of them are symptomatic; so****for pain relief and prevention of adverse events immediate management of such conditions is necessitated (1-6). 98% of small distal ureter stones will be expelled spontaneously and when their diameters are 5 mm, the estimated rate of spontaneous stone passage is 68%, which is 47% for stones > 5 mm and rare for those < 10 mm (5, 7-9). Therefore, active treatments are recommended for patients with larger stones, especially if their stones are larger than 5 mm.

The efficacy of mini invasive therapies such as extracorporeal shock wave lithotripsy (ESWL) and ureteroscopy for distal ureter stones has been proven by recent studies (10). However, these mini invasive therapy modalities have some drawbacks; they are not risk free, sometimes they could even be problematic and also they are quite expensive (11). Moreover the success rate of these techniques is affected by several factors such as stone size and location, machinery type and operator’s experience; in addition, secondary procedures and occasional re-treatments are necessitated (12).

Due to the high rate of multiple treatments and the need for secondary procedures after ESWL, some investigators prefer uretroscopic lithotripsy (URSL), which is a single procedure and has been proven to achieve a higher success rate (13). To facilitate ureteral stone expulsion and decrease post-operative complications, recent studies have recommended a medical expelling therapy (MET) with calcium antagonists, nifedipine, corticosteroids and α-1 blockers (14, 15). Based on these observations, we hypothesized that medical therapy with α-1A blocker after URSL treatment of ureteral stones may increase the success rate and decrease post-operative complications, which may reduce the necessity for a secondary procedure and retreatment.

## 2. Objectives

Therefore we decided to perform a prospective study to evaluate the efficacy of one of the α-1A blockers as an adjunctive therapy after URSL of lower ureteral stones.

## 3. Patients and Methods

This randomized controlled clinical trial was performed at the Urology Department of the Shafa Hospital in the Kerman University of Medical Sciences, Kerman, Iran, from June 2008 to December 2010. The study was explained to all participants, and they could withdraw from the study when they wished. The study was approved by the Ethics Committee of Kerman University of Medical Sciences (Ka/91/285 ethic reference number).

All ureter stone patients who had referred to our clinic (between Jun 2008 to Dec 2010) and had the inclusion criteria were assigned for the study (142 patients). According to the inclusion criteria, all patients had a single and radio opaque lower ureter stone with 5-10 mm diameter, whereas patients with urinary tract infections, high-grade hydronephrosis, diabetes, history of hypersensitivity to α-blockers, ureteral stricture, or pregnant women were excluded. Additionally, we excluded patients with a history of spontaneous stone expulsion, previous ureteral surgery, hypotension or systolic blood pressure < 110 mm Hg.

Stone presence and its characteristics were diagnosed by kidney-ureter-bladder X-ray (KUB), abdominal ultrasonography and intravenous urography (IVU) or helical computed tomography when necessary; stone size was calculated based on the diameter along the ureteral axis before and after the URS and sizes ranged between 5-9 mm.

Those patients that met all the inclusion criteria were enrolled after providing an informed written consent. The operation was performed with general anesthesia. All patients underwent ureteroscopic lithotripsy with a 6 Fr Wolf semi-rigid ureteroscope and a 1.9 Fr pneumatic probe until fragments were smaller than about 2 mm in diameter which allowed for spontaneous passage. Every patient suspected to have complications in lithotripsy procedures (ureteral damages), used temporarily ureteral stent (3 days) and was excluded from the study.

The patients by using block randomization were randomized into intervention and control groups (according to referral time, patients were serially and alternatively placed in "study" and "control" groups), where 52 patients (study group) received tamsulosin (medalocin from Modava Medicine Company) one day before lithotripsy (0.4 mg daily) and 50 patients (control group) received placebo. All patients received their medicine packages blindly from our pharmacists (group A "tamsulosin" and group B "placebo"), so all patients and pharmacy personnel were blinded with regards to the group types and medicine packages. All patients were recommended to drink a minimum of two liters of water per day, and those who had moderate to severe pain (> 5 VAS "visual analogue scale) consumed analgesia (pethedin 25 mg IV, after the procedure in the recovery room and indometacin 500 mg suppository daily). To detect any possible fragments or stone expulsions, all patients were asked to filter their urine. The first assessment of stone clearing rate was performed by imaging during the morning after URSL and then the patients were evaluated at the end of the first and second week after the procedure, with a clinical visit that included KUB and abdominal ultrasonography. Only patients without any residual fragments in KUB and also those who had no signs of hydronephrosis on abdominal ultrasonography were considered to have their stone cleared during the follow-up. The number of colic episodes, lower urinary tract symptoms, amounts of analgesic consumption and adverse effects of medical therapy were recorded in diary and evaluated.

Statistical analysis was carried out with the X^2^ test, Fisher’s exact test and non-parametric Wilcoxon 2-sample t test based on the following parameters: age, stone size, expulsion rate and time, the occurrence of ureteral colic and total analgesic consumption.

## 4. Results

One hundred and two patients completed the study. There were no statistically significant differences between patients of the two groups regarding their demographic findings (Table 1). However, there was a statistically significant difference between the groups regarding expulsion time (P = 0.01), (Table 2). The mean number of colic episodes was 1 ± 0.7 (range 0-2) and 6 ± 3.5 (range 1-9) per patient in the study and control groups, respectively. During the two weeks of study the success rate of stone explosion in both groups was satisfactory, but there was no significant difference between them (X ^2 ^= 1.88, DF = 1, P > 0.1). None of the patients were hospitalized for any post-operative complications (as recurrent colics and urosepsis). Only three patients in the study group experienced adverse effects (transient hypotension, dizziness and palpitations), whereas apart from five patients in the control group, the remaining suffered mild nausea and transient hematuria (P = non-significant). No patient was excluded from the study for these mild complications and the side effects, which disappeared by conservative therapies. Patients who were not stone free after two weeks of follow-up (4 in the study group and 7 in the control group) were successfully re-treated with URSL (Table 3). 

**Table 1.** Patients Characteristics

Characteristic	Study Group (N = 52)	Control Group (N = 50)	P Value
**Age, mean ± SD, y**	24 ± 6.5	27 ± 8.8	0.891
**Men/women **	37/15	40/10	0.812
**Right/left ureter**	27/25	28/22	0.592
**Stone size before URS, mean ± SD [range], mm**	6.6 ± 2.3 [5-9]	6.2 ± 3.2 [4-9]	0.631

**Table 2.** Comparing the Expulsion Time in Study and Control Groups

Expulsion Time, d	Study Group, No. (%)	Control Group, No. (%)	Total, No. (%)	P Value
**< 1**	25 (48.07)	12 (24)	37 (36.27)	0.011
**1-7**	12 (23.07)	13 (26)	25 (24.50)	-
**7-14**	11 (21.15)	10 (20)	21 (20.58)	-
**Stone not passed**	4 (7.69)	15 (30)	19 (18.62)	-

**Table 3.** Results of the Study

Characteristic	Study Group, (n = 52)	Characteristic, (n = 50)	P Value
**Success rate (Expulsion rate)^a^, No. (%)**	49 (93.30)	35 (70)	0.14^b^
**Lower urinary tract symptoms, No. (%)**	7 (13.46)	22 (44)	0.005
**Need for Analgesia (patients), No. (%)**	4 (7.69)	12 (24)	0.032
**Colic episodes, mean ± SD**	1 ± 0.7	6 ± 3.5	0.012
**Mild Complications, No. (%)**	3 (5.76)	5 (10)	0.532^b^

^a^ Success rate (expulsion rate) [X2 = 1.88, DF = 1, P =0.142 (non significant)].

^b^ non-significant.

## 5. Discussion

Recent advances in endourology techniques and new instrumentation largely diverted the treatment of ureteral stones away from open surgery to either minimally invasive methods (e.g. ESWL and URSL), or even watchful waiting; besides, accurate prediction of stone passage may prevent unnecessary intervention and therefore possible complications, especially for the management of distal ureteric stones. The choice of the ideal type of therapy is largely related to the type of equipment available, the type, size, position, degree of impaction and obstruction of the stone, patient preference, and the skills and experiences of the surgeon (16, 17).

Although ureteral stones with a diameter of less than 5 mm could pass for up to 98% of cases, but lithotripsy interventions may cause some degree of ureteral wall congestion and edema that interfere with gravel straight passing and even make stone impaction and obstruction. So the use of MET necessitates for stone passage facilitation and this decreases time for spontaneous passage of gravels, prevents possible risk of renal damage due to prolonged partial ureteral obstruction (greater than 4-6 weeks) and persisting pain or urinary tract infection (7, 18).

In comparison with ESWL, URSL is the optimal and preferred treatment modality for distal ureteric stones (> 5 mm diameter), with URSL allowing direct access to the stones to break them into passable sizes.

Since stone size is the most important factor in all lithotripsy procedures (15), URSL has higher efficacy and success rate, but it is more expensive and more invasive than ESWL (19, 20). Most frequently, complications of URSL occur when the stone gravel is passed, especially through the ureterovesical junction, which is the narrowest part of the ureter. If adjunct therapy such as medical expulsive therapy (MET) is used after lithotripsy or when the ureteroscope is advanced prior to accessing the stone, these complications can be reduced.

The results of our study and other recent studies have demonstrated excellent results for the use of MET for distal ureteral stones. In terms of stone expulsion and control of ureteric colic pain, drugs (e.g. calcium channel blockers, nifedipine, corticosteroids, α1 blockers), that can modulate the function of the ureter that may be obstructed by the stone, can be used. α1 blockers, in particular the α1A blockers such as tamsulosin, are preferred, due to the prevalence of specific adrenoceptor subtype in the distal part of the ureter. Tamsulosin acts by relaxing the ureteral wall muscle and facilitates gravel expulsion after lithotripsy, and also aids with the forwarding of instruments through the ureter for improved stone access.

According to the experiences of Santosh Kumar Singh et al. alpha-blockers efficacy was preferred over the other MET’s efficacies after the ESWL procedure for proximal ureter stones, and they believe that alfa-blockers, especially tamsulosin as a selective sympatholytic agent may reduce complications after stone breaking and gravel passing in all lithotripsy procedures (21-25). In another study Vassilios Tzortzis et al. mentioned that the specific mechanism of action on the ureteral smooth muscle and the emerging evidence about the efficacy (defined as either an increase in expulsion rate or a decrease in time to expulsion) and low-risk profile suggest that α-adrenergic receptor antagonists (α-blockers) and calcium channel antagonists should be the initial preferred METs in distal stone expelling (26). Ureteral colic, associated with obstructing stone or stone gravels after any lithotripsy procedure, increases ureter intraluminal pressure and causes more lactic acid production from smooth muscle spasm and this may have a significant role in stone expelling rate (27). Thus pain relief has an important effect on these events and as we showed in this study, the administration of tamsulosin significantly reduced the need for analgesia in comparison with the control group and this data is in concordance with the other results (24).

Finally, in this case-control study we could not find a significant difference in stone expulsion rate by tamsulosion, which may be due to our small sample size and other simultaneous factors that affect stone passing, such as procedure trauma and its inflammation outcomes and possible urinary infection; thus, for better results we suggest the prescription of tamsulosin along with other METs such as NSADs for subsiding the ureter wall edema which is seen in most obstructing ureter stones and also prophylactic antibiotic prescription for the probable presence of urinary tract infections. Except for the small sample size in this study there was no significant limitation.

Conclusions: Although in this controlled study, we could not find significant differences in the stone expulsion rate by tamsulosion, our study revealed the valuable efficacy of tamsulosin about the low analgesia need and significant low complications in URSL procedure, so we think tamsulosin is a potent α-1-specific blocker with less adverse effects than other α–blockers and components that are used as METs.

However, we should consider the results of this study as preliminary data, which needs to be confirmed by a larger sample size and validated by more extensive investigations in the future.
